# Influence of *TNF* and *IL17* Gene Polymorphisms on the Spondyloarthritis Immunopathogenesis, Regardless of HLA-B27, in a Brazilian Population

**DOI:** 10.1155/2018/1395823

**Published:** 2018-04-19

**Authors:** Marco A. Rocha Loures, Luciana C. Macedo, Denise M. Reis, Camila F. Oliveira, Jean L. Meneguetti, Gabriela F. Martines, Janisleya S. F. Neves, Eliana de Souza, Ana M. Sell, Jeane E. L. Visentainer

**Affiliations:** ^1^Department of Medicine, Maringa State University, Maringá, PR, Brazil; ^2^Post Graduation Program of Biosciences and Physiopathology, Department of Analysis Clinical and Biomedicine, Maringa State University, Maringá, PR, Brazil; ^3^Laboratory of Immunogenetics, Department of Basic Health Sciences, Maringa State University, Maringá, PR, Brazil

## Abstract

**Background and Objectives:**

Spondyloarthritis (SpA) represents a heterogeneous group of immune-mediated inflammatory diseases that have overlapping clinical features, genetic predisposition, and pathogenic mechanisms. Hence, we investigated, through a case-control study, whether single-nucleotide polymorphisms of *TNF* and *IL17* genes are associated with SpA, ankylosing spondylitis (AS), and psoriatic arthritis (PsA) in a mixed Brazilian population.

**Methods:**

Genotyping of *TNF*-308 (rs1800629), *TNF*-238 (rs361525), *IL17A* (rs2275913), *IL17F* (rs763780), and *HLA-B27* polymorphisms was performed in 243 patients with SpA and 210 controls from Southern Brazil using SSOP-Luminex (One Lambda) and PCR-SSP assays.

**Results:**

Significant associations were confirmed between the HLA-B27 marker and SpA, AS, and PsA diseases. While *TNF*-308 (rs1800629) AA/GA, *IL17A* (rs2275913) AA/GA, and *IL17F* (rs763780) CC/TC genotype frequencies were associated, in the dominance inheritance model, with SpA and AS, regardless of gender, the presence of *HLA-B27*, *TNF*-238 (rs361525) GA/AA, *IL17A* (rs2275913) AA/GA, and *IL17F* (rs763780) genotypes was associated with PsA.

**Conclusion:**

In this Brazilian population, *TNF* and *IL17* gene polymorphisms responsible for the expression of important inflammatory cytokines were associated with overall SpA, and, specifically, with AS and PsA, regardless of gender and *HLA-B27*. However, future larger studies with different ethnicities may be necessary to confirm these genetic associations.

## 1. Introduction

Spondyloarthritis (SpA) represents a heterogeneous group of immune-mediated inflammatory diseases that have overlapping clinical features, genetic predisposition, and pathogenic mechanisms. The disease may be undifferentiated or manifest as reactive arthritis, psoriatic arthritis (PsA), arthritis associated with inflammatory bowel disease, or ankylosing spondylitis (AS) [1, 2].

The pathogenesis of SpA is partly attributed to the interaction between genetic factors and the environment. Males are affected more frequently than females according to results from two meta-analysis studies [3, 4], and genetic factors associated with HLA in the pathogenesis of SpA are best represented by the strong association between the *HLA-B27* and AS [4]. However, the fact that only 1–3% of *HLA-B27*-positive people develop the disease, and not all patients with the disease possess the HLA-B27 antigen, suggests that other genes may be involved in the development of the pathology [5, 6].

Polymorphisms in genes encoding cytokines that interfere on cytokine level production have been associated with rheumatologic chronic inflammatory diseases [7–10]. However, the role of the tumor necrosis factor (*TNF*) and interleukin (*IL*) *17A* and *IL17F* genes, encoded on the same chromosome 6 of the *HLA* genes, and their clinical importance in the pathogenesis of SpA have not been fully elucidated [11–13]. Proinflammatory cytokines such as TNF-*α* and IL17 may be elevated in patients with SpA and may contribute to the pathogenesis of the disease (reviewed by Zambrano-Zaragoza et al.) [11]. These cytokines have cytotoxic effects and induce the secretion of other cytokines, such as TNF-*α*, IL1*β*, and IL6, which can cause generalized tissue damage [14].

Thus, the aim of the present study was to investigate whether the single-nucleotide polymorphisms (SNPs) of the *TNF*, *IL17A*, and *IL17F* genes are associated with SpA and its clinical forms, AS, and PsA, in a mixed population from the South of Brazil.

## 2. Patients and Methods

### 2.1. Patients and Controls

In this case-control study, 243 unrelated patients were diagnosed by the ASAS Criteria 2009 [14] for axial SpA and the ASAS Criteria 2011 [15] for peripheral SpA. All patients had magnetic resonance imaging of the sacroiliac joints and were evaluated for the presence of *HLA-B27*. In patients with PsA, we also used the CASPAR criteria [16], to complement the ASAS criteria, to provide greater security in the data obtained. Patients were selected at the Maringa University Hospital-Maringa State University and the Maringa Rheumatism Clinic, from Paraná State, Southern Brazil.

In addition, 210 individuals were selected to form the control group, following the criteria for inclusion: no autoimmune and/or rheumatic diseases, unrelated to the patient group, and belonging to the same ethnic group as the patients.

The population of Paraná is predominantly of European origin (80.6%), with a small but significant contribution of African (12.5%) and Amerindian (7.0%) genes [17]. Both the patients and healthy controls were classified as mixed ethnic groups, according to phenotypic characteristics, because according to Parra et al. [18], in Brazil, at an individual level, skin color determined by physical evaluation is a poor predictor of genomic African ancestry.

The project was approved by the Ethics Committee of the Maringa State University (number 687.222/2014). After terms of consent were signed, blood samples were collected, centrifuged, and frozen at −20°C until use.

### 2.2. Analysis of Genetic Cytokine Polymorphisms

Genotyping of *TNF*-238 (rs361525), *TNF*-308 (rs1800629), *IL17A* (rs2275913), and *IL17F* (rs763780) SNPs was performed with DNA samples by PCR-restriction fragment length polymorphism (RFLP) [19, 20]. Primer sequences, conditions, restriction enzymes, and size fragments are shown in Table 1.

### 2.3. Identification of HLA-B27

Genotyping of HLA-B27 was performed using PCR-SSP according to the method published before by our research group [21].

### 2.4. Statistical Analysis

The software Quanto (http://biostats.usc.edu/Quanto.html) was used to calculate the sample size using the less frequent allele (0.095 for *TNF*-238), population risk (1.5%), and OR (2.0–4.0).

The allele and genotype frequencies in patients and controls were estimated and compared by chi-square distribution tables using the OpenEpi 3.01 software (http://www.openepi.com/Menu/OE_Menu.htm). The statistical comparisons between groups were realized, and the estimated risk in individuals who hold genetic polymorphisms was calculated by the determination of OR (odds ratio) with 95% of confidence interval, adjusted for gender and age. All statistical analyses were performed by the software SNPStats (http://bioinfo.iconcologia.net/index.php), which was also used for detecting the Hardy-Weinberg (HW) and odds ratio balance, with a 95% confidence interval (CI). The *P* ≤ 0.05 values were considered statistically significant to the chi-square test with Yates' correction and logistic regression. The association tests were realized to the codominant, dominant, recessive, overdominant, and log-additive genetic inheritance models.

Finally, because *TNF*-238, *TNF*-308, *IL17A*, and *IL17F* genes are located near chromosome 6, we performed a haplotype analysis for those SNPs associated with SpA, AS, and PsA, with the aim of establishing the disease risks if the individual possesses determined haplotype using the software SNPStats.

## 3. Results

Baseline characteristics of patients (*n* = 243) and controls (*n* = 210) regarding age, gender, and genetic marker HLA-B27 are presented in Table 2. The majority of them were females (55.2% of patients and 56.2% of controls), with an average age of 47 (±15.7) and 40 (±2.7) years, respectively. The HLA-B27-positive marker was present, in a higher percentage, in patients with SpA than in controls (36.6% versus 15.2%) and in patients with AS and PsA than in controls (40.8% and 26.9%, resp., versus 15.2%).

Allele and genotype polymorphisms for *TNF*-308, *TNF*-238, *IL17A*, and *IL17F* in patients and healthy controls are presented in the Supplementary Table available here. The distribution of the genotypic frequencies of these polymorphisms in controls was in HW equilibrium.

Multivariate analysis after adjustment for gender and the presence of the *HLA-B27* marker revealed an independent effect of SpA susceptibility in the possession of such genotypes: *TNF*-308 GA/AA, *IL17A* GA/AA, and *IL17F* TC/CC, with an OR (95% CI) = 1.7 (1.1–2.5), 1.7 (1.2–2.6), and 4.5 (2.6–7.6), respectively (Table 3).

In patients with AS, there was a significant difference between *TNF*-308 GA/AA, *IL17A* GA/AA, and *IL17F* TC/CC genotypes, in the dominant model, independent of gender and HLA-B27 (Table 4).

Our analysis also showed the association between the PsA and *TNF*-238 GA/AA, *IL17A* GA/AA, and *IL17F* TC/CC genotypes, in the dominant model, regardless of both gender and HLA-B27 marker (Table 5).

In addition, there were statistical differences in haplotype distribution between patients and controls, independent of HLA-B27, for developing SpA, AS, and PsA if the patient possesses the haplotypes *TNF*-308/*IL17A*/*IL17F* GGC, GAC, and AAT (Table 6).

## 4. Discussion

In this study, we investigated the association of genetic polymorphisms relative to TNF-*α*, IL17A, and IL17F cytokines, of particular interest in the inflammation seen in SpA [5], with the development of the disease and its clinical forms (AS and PsA) in a mixed Brazilian population. The results point genotypes and alleles of *TNF*-238, *TNF*-308, *IL17A*, and *IL17F* as risk factors for these diseases.

Accumulating evidence has demonstrated that HLA-B27 is strongly related to SpA inducing immune inflammatory responses, especially in AS patients [4–6]. In our current study, we confirmed these findings for SpA (36.2%), AS (59.8%), and PsA (26.9%) versus controls (15.7%).

Recently, a meta-analysis suggested a positive association between HLA-B27 and sex (male) in AS patients [4]. Then, we evaluated the risk of the inflammatory cytokine gene polymorphisms on the development of SpA, AS, and PsA diseases, considering the gender and the presence of the HLA-B27 marker.

In our current study, we found *TNF* and *IL17* genotypes associated with these diseases, independent of these factors, which could provide more powerful evidence of association between these polymorphisms and SpA, AS, and PsA susceptibility.

In numerous studies, *TNF*-308 and *TNF*-238 polymorphisms were considered to be factors involved in SpA pathogenesis, although other groups failed to confirm this theory [22, 23]. Potential reasons for this disagreement are the different ethnicities of the populations studied, the low number of patients and controls, the lack of pairing criteria in the control group, and the noncompliance with other risk factors.

Hence, in this study, care was taken with the pairing of patients and controls, regarding age, sex, and ethnicity. The control group included individuals of the minimum age at which disease develops and with the same proportion of men and women, since some forms of the disease may be more common in a specific gender. To minimize stratification errors due to differences in allelic frequencies in different ethnic groups, only patients and controls belonging to the mixed ethnic groups (descendants of immigrant Europeans, native Amerindians, and Africans) were included.

In *TNF*, *IL17A*, and *IL17F* genes, mapped on the same chromosome at position 6p12, several polymorphic sites, including microsatellites and promoter polymorphisms, have been detected, which may influence the expression of TNF-*α*, IL17A, and IL17F. The substitution of G for A at the −308 position of the *TNF* gene promoter leads to increased production of TNF-*α*, whereas the substitution of G by A at position −238 results in decreased expression [24–26]. Previous studies also show that genetic polymorphisms of *IL17A* G197A and *IL17F* T7488C affect the production of IL17A and IL17F, respectively [27, 28].

Our analysis showed that the *TNF*-308 GA/AA genotype was associated with the development of overall SpA and AS, independent of gender and HLA-B27 allelic group. This finding is in agreement with that of Höhler et al. who demonstrated an association of the *TNF*-308 A/A genotype with AS in German patients [29] and that of Romero-Sánchez et al. who showed that the A allele frequency of *TNF*-308 was increased in Columbian patients with SpA, AS, and rheumatoid arthritis [30]. This suggests that *TNF*-308A could be a susceptibility factor for this disease.

We have also found an association between the *TNF*-238 GA/AA genotypes and PsA in concordance to Rahman et al. [31] who examined the association between the *TNF* promoter gene polymorphisms and psoriatic arthritis in two well-characterized Canadian populations with the disease and carried out a meta-analysis of all *TNF* association studies in white populations with psoriatic arthritis.

However, a meta-analysis of 2247 Korean patients with AS showed no association of *TNF*-308 AA and AA/AG or *TNF*-238 A/G polymorphisms with the disease [23]. One hypothesis for this disagreement would be the ethnic admixture found among populations, since our study did not include Asian descendants.

In addition, we observed a higher risk of SpA in patients carrying the *IL17A* A/G genotype and A allele and the *IL17F* T/C genotype and C allele. To the best of our knowledge, an association of these genetic polymorphisms with SpA has not yet been described. Our group already reported that the AA genotype and A allele for *IL17A* were associated with susceptibility to chronic periodontitis in South Brazil, highlighting their involvement in a chronic inflammatory condition [18]. A weak association between rheumatoid arthritis and the promoter *IL17A* rs2275913 was found in the Norwegian population and more recently in the Brazilian population [7, 32]. Specifically, with the risk of AS and its severity, a strong association was observed with the rs4819554 SNP in the promoter region of *IL17RA* in two Spanish cohorts of patients and controls, but not involving the *IL17A* and *IL17F* polymorphisms that we have studied here [33]. Therefore, in patients of Northern Italy, *IL17A* and *IL17RA* gene allelic variants were not associated with PsA susceptibility [34].

In a study of 371 patients, Baeten et al. showed that treatment with anti-IL17 medication in both subcutaneous and intravenous forms led to an improvement in the clinical picture [35]. This provided evidence for the role of increased IL17 production in disease development. In another study, Berg and McInnes also demonstrated a good therapeutic response in 606 PsA patients treated with anti-IL17 medication [36]. The recent use of this inhibitor led to a significant and sustained reduction in the signs and symptoms of PsA, avoiding radiological progression and thereby improving patients' quality of life [37].

Relevant result of this study was establishing risks to the development of SpA, AS, and PsA, related to the haplotype that the individual possesses for these cytokine SNPs on chromosome 6, regardless of *HLA-B27*. The risks were higher in patients with the haplotypes *TNF*-308/*IL17A*/*IL17F* GGC/GAC/AAT for SpA and AS and *TNF*-238/*IL17A*/*IL17F* GGC/GAC/AAT for PsA than in those who did not have. We may suggest that in the presence of the C allele for *IL17F*, the A allele for *TNF*-308 and *IL17A* is not required for a positive association with the diseases. However, if the A allele is present for both, the C allele for *IL17F* is not needed.

Previous findings have suggested *IL17* and *TNF* haplotypes involved in the risk of developing obstructive coronary artery disease [38, 39], which has been reported in patients with SpA [40]. This could explain our findings involving cytokine gene polymorphisms associated with inflammatory responses observed in these patients suffering from rheumatic diseases.

Finally, these observations should be interpreted with caution due to limitations found in this study, such as the relatively small sample size and the fact that only the *TNF* and *IL17* genes were considered in this study. Moreover, we did not evaluate the possibility of interactions between these genes and other genetic risk factors, beyond the gender and *HLA-B27*, known to interfere in the development of SpA.

## 5. Conclusion

In conclusion, in this mixed Brazilian population, *TNF*-308, *IL17A*, and *IL17F* gene polymorphisms were associated with SpA and AS, while *TNF*-238, *IL17A*, and *IL17F* gene polymorphisms were associated with PsA, regardless of gender and *HLA-B27*. The elucidation of the role of these biological markers together with the HLA-B27 marker, in the development of inflammatory diseases, in larger series of subjects, could provide the early diagnosis and treatment of the different clinical forms of this disease.

## Figures and Tables

**Table 1:** Primer sequences, conditions, restriction enzymes, and size fragments.

*TNF* and *IL17* SNPs	Primer sequences	Conditions	Restriction enzymes	Size fragments
*TNF-238* (rs361525)	5-ATCTGGAGGAAGCGGTAGTG-3 5-AGAAGACCCCCCTCGGAACC-3	94°C—5 min	MspI	107 pb
		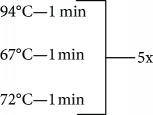		87 + 20 pb
		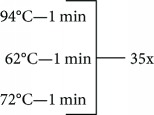		
		72°C—7 min		
		4°C—∞		
*TNF-308* (rs1800629)	5-AGGCAATAGGTTTTGAGGGGCCAT-3 5-TCCTCCCTGCTCCGATTCCG-3	94°C—5 min	NcoI	152 pb
		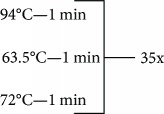		133 + 19 pb
		72°C—7 min		
		4°C—∞		
*IL17A-G197A* (rs2275913)	5-AACAAGTAAGAATGAAAAGAGGACATGGT-3 5-CCCCCAATGAGGTCATAGAAGAATC-3	95°C—5 min	EcoNI	68 + 34 pb
		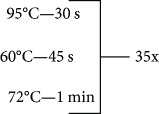		102 + 68 + 34 pb
				102 pb
		72°C—10 min		
		4°C—∞		
*IL17F-T7488C* (rs763780)	5-ACCAAGGCTGCTCTGTTTCT-3 5-GGTAAGGAGTGGCATTTCTA-3	96°C—5 min	NlaIII	63 + 80 pb
		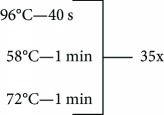		143 + 80 + 63 pb
				143 pb
		72°C—7 min		
		4°C—∞		

**Table 2:** Baseline characteristics of patients and healthy volunteers (controls).

	SpA (*N* = 243)	AS (*N* = 174)	PsA (*N* = 67)	Controls (*N* = 210)
Mean age (SD) years	47 (±15.7)	45 (±15.3)	51 (±16.1)	40 (±2.7)
Gender, males [*n* (%)]	109 (44.9)	81 (46.6)	28 (41.8)	92 (43.8)
Gender, females [*n* (%)]	134 (55.2)	93 (53.6)	39 (58.2)	118 (56.2)
*HLA-B*∗27 positive [*n* (%)]	89 (36.6)^a^	71 (40.8)^b^	18 (26.9)^c^	32 (15.2)^a,b,c^

SpA: spondyloarthritis; AS: ankylosing spondylitis; PsA: psoriatic arthritis. Statistical analysis was performed by chi-square distribution table using OpenEpi 3.01 software: ^a^ *P* < 0.001, OR (95% CI) = 3.2 (2.0–5.0). ^b^ *P* < 0.001, OR (95% CI) = 8.3 (5.1–13.4). ^c^ *P* < 0.03, OR (95% CI) = 2.0 (1.1–4.0).

**Table 3:** Association of *TNF-308*, *IL17A*, and *IL17F* with the risk of spondyloarthritis, stratified by gender and *HLA-B27* marker.

Gene (SNP)	Allele genotypes	SpA (*N* = 243) [*n* (%)]	Controls (*N* = 210 [*n* (%)]	*P* value^1^	OR (95% CI)
*TNF-308* (rs1800629)	G/G	144 (59.3)	150 (71.4)		
	G/A	95 (39.1)	58 (27.6)		
	A/A	4 (1.6)	2 (1.0)		
	G/A + A/A	99 (40.7)	60 (28.6)	0.01	1.7 (1.1–2.5)

		(*N* = 243)	(*N* = 210)		
*IL17A* (rs2275913)	G/G	114 (46.9)	123 (58.6)		
	G/A	102 (42.0)	71 (33.8)		
	A/A	27 (11.1)	16 (7.6)		
	G/A + A/A	129 (53.1)	87 (41.4)	0.005	1.7 (1.2–2.6)

		(*N* = 242)	(*N* = 209)		
*IL17F* (rs763780)	T/T	161 (66.5)	188 (90.0)		
	T/C	76 (31.4)	20 (9.6)		
	C/C	5 (2.1)	1 (0.5)		
	T/C + C/C	81 (33.5)	21 (10.1)	<0.001	4.5 (2.6–7.6)

SNP: single-nucleotide polymorphism; SpA: spondyloarthritis; OR: odds ratio; CI: confidence interval. ^1^Statistical analysis was performed by SNPStats software based on a statistical dominant model.

**Table 4:** Association of *TNF-308*, *IL17A*, and *IL17F* with the risk of AS, stratified by gender and *HLA-B27* marker.

Gene (SNP)	Allele genotypes	AS (*N* = 172) [*n* (%)]	Controls (*N* = 210) [*n* (%)]	*P* value^1^	OR (95% CI)
*TNF-308* (rs1800629)	G/G	95 (55.2)	150 (71.4)		
	G/A	74 (43.0)	58 (27.6)		
	A/A	3 (1.7)	2 (1.0)		
	G/A + A/A	77 (44.8)	60 (28.6)	0.004	1.9 (1.2–3.0)

		(*N* = 172)	(*N* = 210)		
*IL17A* (rs2275913)	G/G	81 (47.1)	123 (58.6)		
	G/A	74 (43.0)	71 (33.8)		
	A/A	17 (9.9)	16 (7.6)		
	G/A + A/A	91 (52.9)	87 (41.4)	0.01	1.7 (1.1–2.6)

		(*N* = 171)	(*N* = 210)		
*IL17F* (rs763780)	T/T	119 (66.6)	188 (90.0)		
	T/C	48 (28.1)	20 (9.6)		
	C/C	4 (2.3)	1 (0.5)		
	T/C + C/C	52 (30.4)	21 (10.1)	<0.001	3.7 (2.1–6.6)

SNP: single-nucleotide polymorphism; AS: ankylosing spondylitis; OR: odds ratio; CI: confidence interval. ^1^Statistical analysis was performed by SNPStats software based on a statistical dominant model.

**Table 5:** Association of *TNF*-238, *IL17A*, and *IL17F* with the risk of PsA, stratified by gender and *HLA-B27* marker.

Gene (SNP)	Allele genotypes	PsA (*N* = 67) [*n* (%)]	Controls (*N* = 210) [*n* (%)]	*P* value^1^	OR (95% CI)
*TNF-238* (rs361525)	G/G	50 (74.6)	181 (86.2)		
	G/A	16 (23.9)	28 (13.3)		
	A/A	1 (1.5)	1 (0.5)		
	G/A + A/A	17 (25.4)	29 (13.8)	0.02	2.3 (1.1–4.5)

		(*N* = 67)	(*N* = 210)		
*IL17A* (rs2275913)	G/G	30 (44.8)	123 (58.6)		
	G/A	27 (40.3)	71 (33.8)		
	A/A	10 (14.9)	16 (7.6)		
	G/A + A/A	37 (55.2)	87 (41.4)	0.02	1.9 (1.1–3.4)

		(*N* = 67)	(*N* = 209)		
*IL17F* (rs763780)	T/T	40 (59.7)	188 (90.0)		
	T/C	26 (38.8)	20 (9.6)		
	C/C	1 (1.5)	1 (0.5)		
	T/C + C/C	27 (40.3)	21 (10.1)	<0.001	6.6 (3.3–13.1)

SNP: single-nucleotide polymorphism; PsA: psoriatic arthritis; OR: odds ratio; CI: confidence interval. ^1^Statistical analysis was performed by SNPStats software based on a statistical dominant model.

**Table 6:** Distribution of *TNF-308/IL17A/IL17F* haplotypes in individuals with SpA, AS, and PsA (interaction analysis with gender covariate).

Haplotype	SpA	AS	PsA
*TNF-308/IL17A/IL17F*	OR (CI 95%)	OR (CI 95%)	OR (CI 95%)
GGC	3.9 (1.7–9.0)	2.5 (1.1–5.7)	7.0 (2.8–17.7)
GAC	5.0 (2.1–12.0)	4.1 (1.6–10.3)	5.5 (1.4–22.1)
AAT	4.2 (1.6–11.3)	4.4 (1.5–13.1)	5.2 (1.1–23.7)

SpA: spondyloarthritis; AS: ankylosing spondylitis; PsA: psoriatic arthritis; OR: odds ratio; CI: confidence interval.

## Supplementary Materials

Supplementary Materials Supplementary Table: genotype and allele frequencies of *TNF*-238, *TNF*-308, *IL17A*, and *IL17F* in patients with SpA, AS, and PsA and in controls are shown in this material.
