# Supplementary material for: Influence of TNF and IL17 Gene Polymorphisms on the Spondyloarthritis Immunopathogenesis, Regardless of HLA-B27, in a Brazilian Population
Source: Mediators Inflamm. 2018 Apr 19;2018:1395823. doi: 10.1155/2018/1395823 (PMC5933012; doi:10.1155/2018/1395823)
Supplement: Supplementary Materials — Supplementary Table: genotype and allele frequencies of TNF-238, TNF-308, IL17A, and IL17F in patients with SpA, AS, and PsA and in controls are shown in this material. [file 1395823.f1.pdf]

Table Supplementary. Genotype and allele frequencies of *TNF*-238, *TNF*-308, *IL17A* and *IL17F* in patients with SpA, AS, PsA and in controls

Gene and SNP	Allele Genotypes	SpA (243) n (%)	AS (173) n (%)	PsA (67) n (%)	Controls (210) n (%)
TNF-238	G	440 (90.5)	319 (92.2)	116 (86.6)	390 (92.9)
(rs361525)	A	46 (9.5)	27 (7.8)	18 (13.4)	30 (7.1)
	G/G	198 (81.5)	146 (84.4)	50 (74.6)	181 (86.2)
	G/A	44 (18.1)	27 (15.6)	16 (23.9)	28 (13.3)
	A/A	1 (0.4)	0 (0)	1 (1.5)	1 (0.5)
		N = 243	N = 173	N = 67	N = 210
TNF-308	G	383 (78.8)	266 (76.9)	111 (82.8)	358 (85.2)
(rs1800629)	A	103 (21.2)	80 (23.1)	23 (17.2)	62 (14.8)
	G/G	144 (59.3)	96 (55.5)	45 (67.2)	150 (71.4)
	G/A	95 (39.1)	74 (42.8)	21 (31.3)	58 (27.6)
	A/A	4 (1.6)	3 (1.7)	1 (1.5)	2 (1.0)
		N = 243	N = 173	N = 67	N = 210
IL17A	G	330 (67.9)	238 (68.8)	87 (64.9)	317 (75.5)
(rs2275913)	A	156 (32.1)	108 (31.2)	47 (35.1	103 (24.5)
	G/G	114 (46.9)	82 (47.4)	30 (44.8)	123 (58.6)
	G/A	102 (42.0)	74 (42.8)	27 (40.3)	71 (33.8)
	A/A	27 (11.1)	17 (9.8)	10 (14.9)	16 (7.6)
		N = 242	N = 172	N = 67	N = 210
IL17F	T	398 (82.2)	288 (83.7)	106 (79.1)	398 (94.8)
(rs763780)	C	86 (17.8)	56 (16.3)	28 (20.9)	22 (5.2)
	T/T	161 (66.5)	120 (69.8)	40 (59.7)	189 (90.0)
	T/C	76 (31.4)	48 (27.9)	26 (38.8)	20 (9.5)
	C/C	5 (2.1)	4 (2.3)	1 (1.5)	1 (0.5)

SpA, Spondyloarthritis; AS, Ankylosing Spondylitis; PsA, Psoriatic Arthritis.
